# Effect of argon cold plasma composition on orthodontic bonding—new insights into input parameters and protocols

**DOI:** 10.1007/s00056-023-00451-9

**Published:** 2023-03-01

**Authors:** Mostafa M. Alzahar, Karl-Friedrich Krey, Philine H. Doberschütz

**Affiliations:** 1 Department of Orthodontics, Greifswald University Medicine, Greifswald, Germany; 2 German National Center for Plasma Medicine (NZPM), Berlin, Germany

**Keywords:** Plasma gases, Dental enamel, Dental acid etching, Dental bonding, Orthodontic brackets, Plasmagase, Zahnschmelz, Dentales Säureätzen, Dentales Bonding, Kieferorthopädische Brackets

## Abstract

**Purpose:**

Cold atmospheric plasma can functionalize enamel without damaging the substrate morphology. It therefore has the potential to be a gentle alternative to conventional acid etching. To realize the full potential of cold atmospheric plasma in orthodontic bonding, the input parameters and protocols that are most beneficial to surface modification must first be identified. We aimed to clarify how the admixture of oxygen to cold atmospheric plasma and the rewetting of the enamel affect the conditioning properties for orthodontic bonding.

**Methods:**

First, we illustrated the morphological effects of different plasma compositions on the enamel surface by means of scanning electron microscopy. Then, we measured the shear bond strength resulting from different conditioning techniques on bovine enamel specimens: conventional acid etching; no conditioning; pure argon plasma; argon plasma plus 0.5% oxygen admixture; argon plasma plus 0.5% oxygen and rewetting after plasma application. Brackets were bonded using light cured adhesive; all specimens were subjected to thermocycling. The shear bond strength of each specimen was measured in a universal testing machine and compared using Welch one-way analysis of variance (ANOVA) and Games–Howell post hoc test.

**Results:**

Specimens conditioned with argon plasma plus 0.5% oxygen and rewetting showed a significantly higher shear bond strength than specimens conditioned with conventional acid etching. Conditioning with pure argon plasma and argon plasma plus 0.5% oxygen without rewetting yielded significantly lower shear bond strength.

**Conclusion:**

Admixing 0.5% oxygen and rewetting the enamel after plasma application are crucial steps that could help make cold atmospheric plasma a gentle conditioning technique in orthodontic bonding.

## Abstract (de)

**Zweck:**

Kaltes Argonplasma kann die Oberflächenenergie und Benetzbarkeit von Zahnschmelz erhöhen, ohne ausgeprägte Veränderungen oder Schäden in der Substratmorphologie hervorzurufen. Um dieses Potenzial in der Kieferorthopädie nutzen zu können, müssen zunächst die Parameter und Protokolle identifiziert werden, die für den Einsatz von Adhäsivsystemen am vorteilhaftesten sind. Unsere Studie untersucht, wie die Beimischung von Sauerstoff zu Argonplasma und die Wiederbenetzung des Schmelzes die konditionierenden Eigenschaften für das kieferorthopädische Bonding beeinflussen.

**Methoden:**

Mittels Rasterelektronenmikroskopie wurden zunächst die morphologischen Auswirkungen verschiedener Plasmazusammensetzungen auf die Schmelzoberfläche dargestellt. Anschließend wurden Rinderschmelzproben mit verschiedenen Protokollen konditioniert: herkömmliches Säureätzen; keine Konditionierung; reines Argonplasma; Argonplasma plus 0,5 % Sauerstoffbeimischung; Argonplasma plus 0,5 % Sauerstoffbeimischung und Wiederbenetzung nach Plasmaauftrag. Die Brackets wurden mit lichthärtendem Adhäsiv befestigt und alle Proben einem Thermocycling unterzogen. Die Scherhaftfestigkeit jeder Probe wurde in einer Universalprüfmaschine gemessen und mittels Welch-Einweg-ANOVA („analysis of variance“) und Games-Howell-Post-Hoc-Test verglichen.

**Ergebnisse:**

Proben, die mit Argonplasma plus 0,5 % Sauerstoff und Wiederbenetzung konditioniert wurden, zeigten eine signifikant höhere Scherhaftfestigkeit als Proben, die mit herkömmlichem Säureätzen konditioniert wurden. Die Konditionierung mit reinem Argonplasma und Argonplasma plus 0,5 % Sauerstoff ohne Wiederbenetzung ergab eine signifikant niedrigere Scherhaftfestigkeit.

**Schlussfolgerung:**

Die Beimischung von 0,5 % Sauerstoff und die Wiederbenetzung des Zahnschmelzes nach der Plasmaanwendung sind entscheidende Schritte, die dabei helfen könnten, kaltes Argonplasma zur substratschonenden Konditionierung im kieferorthopädischen Bonding nutzen zu können.

## Introduction

An optimal bonding technique provides adequate adhesive strength while being gentle on the dental hard tissue. Substance conservation is especially important in orthodontics, because unlike in other disciplines of dentistry, bonding for orthodontic purposes is intended to be temporary: After the active treatment, all brackets are removed—ideally without leaving visible traces or damaging the enamel surface. Despite this, similar techniques and materials are used for orthodontic bonding as for dental restorations designed for a much longer lifespan. These common composite bonding systems rely on micromechanical interlocking by infiltrating into the microporosities of acid-etched enamel. The accompanying irreversible substance loss has long been criticized [17]. By dissolving the fluoride-rich superficial enamel layer, acid etching can increase the risk of decalcification [11], which in turn can negatively impact smile esthetics, particularly in the incisor region. Developing a gentle alternative to acid etching is therefore of great interest for orthodontics.

In recent years, cold atmospheric plasma (CAP) has received increasing scientific attention due to its surface modifying properties. CAP—excited gas containing charged and reactive particles—can functionalize enamel without compromising the morphology [12]. By depositing active species on the tooth, CAP can increase the surface energy and wettability [6, 21]. The gas temperature of less than 40 °C (104 °F) allows for safe application on dental enamel. CAP therefore presents the potential to replace or supplement conventional acid etching, as it has been proposed for dental restorations [5] and orthodontic brackets [1, 14].

While most plasma devices in preclinical and clinical use utilize pure argon gas for CAP generation, current research shows that the characteristics of the plasma effluent can be modified by admixing oxygen to the feed gas [2, 3]. Regarding orthodontics, this raises the question which CAP composition is most suitable for bracket bonding. As Bekeschus et al. [3] emphasize in their recently published paper, the influence of feed gas modulation on the biological results of CAP application, has not yet been sufficiently researched.

It is also of interest to investigate whether the enamel should be rewetted after CAP application: The plasma effluent is a focused stream of excited gas and as such dries out the surface it is applied to. Results from our previous study have indicated that rewetting the dry enamel surface after CAP application can increase the shear bond strength [14].

Thus, to realize the full potential of CAP in orthodontic bonding, the input parameters and protocols that are most beneficial to surface modification must first be identified. We aimed to clarify how the admixture of oxygen and the rewetting of the enamel surface after CAP application affect the conditioning properties of argon CAP for orthodontic bonding. In a prestudy, we used scanning electron microscopy to determine how the admixture of varying amounts of oxygen to argon CAP affects the morphological integrity of human and bovine enamel. In our main study, we then compared the shear bond strength of brackets bonded on dry and rewetted bovine enamel conditioned with pure argon CAP and argon CAP with 0.5% oxygen admixture, with the shear bond strength on acid-etched enamel.

## Materials and methods

### Prestudy: Illustrating the morphological effects caused by different CAP compositions

Bovine mandibular incisors were obtained from a commercial provider (Rocholl, Aglasterhausen, Germany) officially registered with the veterinary authority (EU approval code: DE 08 2 25 0001 14). Human molar teeth were obtained from the Department of Oral Surgery, University Medicine Greifswald, directly after extraction.

Storage and processing of all specimens followed the recommendations of ISO/TS11405:2015(E) [9]. After disinfection (10% chloramine‑T hydrate solution), the flattest buccal portion of each specimen was identified, cut into a square block, and attached to a scanning pin. All specimens were cleaned with an oil-free, nonfluoridated paste (Super Polish, KerrHawe SA, Bioggio, Switzerland; REA 4.5, 15 s, 6000 rpm) and thoroughly rinsed with water (15 s).

Specimens were divided into five groups with five bovine and five human specimens each:

- 1) Specimens were conditioned using a standard etch-and-rinse system: acid etching (Transbond XT, 3M, Saint Paul, MN, USA; 30 s, 35% phosphoric acid), water rinsing (20 s), air drying (20 s, oil-free air spray).
- 2) Specimens were conditioned using pure argon CAP (kINPen MED, neoplas tools GmbH, Greifswald, Germany); treatment time was 30 s per specimen.
- 3) Specimens were conditioned using argon CAP with 0.5% oxygen admixture (kINPen MED, neoplas tools GmbH, Greifswald, Germany); treatment time was 30 s per specimen.
- 4) Specimens were conditioned using argon CAP with 1% oxygen admixture (kINPen MED, neoplas tools GmbH, Greifswald, Germany); treatment time was 30 s per specimen.
- 5) Specimens were left untreated after polishing.

Directly after treatment, the specimens were sputter-coated with Au–Pd alloy (100 s; SCD050, Leica Microsystems, Wetzlar, Germany) and the surface modifications were subjected to morphological visualization by scanning electron microscopy (Zeiss evo LS 10, Carl Zeiss Microscopy, Jena, Germany). These specimens were discarded after imaging and were not used for shear bond strength testing. Representative images were selected from each group to be shown as figures in this publication.

### Main study: Determining the shear bond strength

Bovine mandibular incisors were obtained from a commercial provider (Rocholl, Aglasterhausen, Germany) officially registered with the veterinary authority (EU approval code: DE 08 2 25 0001 14). Storage and processing of all specimens followed the recommendations of ISO/TS11405:2015(E) [9]. After disinfection (10% chloramine‑T hydrate solution), all teeth were embedded in cylindrical blocks of resin (Technovit 4071, Heraeus Kulzer, Wehrheim, Germany), exposing the facial surface. An area of low labial curvature (5 mm × 5 mm), was marked on the teeth, cleaned with an oil-free, nonfluoridated paste (Super Polish, KerrHawe SA, Bioggio, Switzerland; REA 4.5, 15 s, 6000 rpm), thoroughly rinsed with water (15 s) and dried using an oil- and moisture-free air source.

Specimens were divided into five groups:

- Group I (*n* = 25): the enamel was conditioned using a standard etch-and-rinse system: acid etching (Transbond XT, 3M, Saint Paul, MN, USA; 30 s, 35% phosphoric acid), water rinsing (20 s), air drying (20 s, oil-free air spray).
- Group II (*n* = 25): the enamel was not conditioned prior to the application of the primer.
- Group III (*n* = 25): the enamel was conditioned using pure argon CAP (kINPen MED, neoplas tools, Greifswald, Germany); treatment time was 30 s per specimen.
- Group IV (*n* = 25): the enamel was conditioned using argon CAP with 0.5% oxygen admixture (kINPen MED, neoplas tools, Greifswald, Germany); treatment time was 30 s per specimen.
- Group V (*n* = 25): the enamel was conditioned using argon CAP with 0.5% oxygen admixture (kINPen MED, neoplas tools, Greifswald, Germany); treatment time was 30 s per specimen; after CAP application the enamel was rewetted with deionized water.

Brackets (Mini-mono, Forestadent, Pforzheim, Germany; tooth number 8) were bonded on all specimens using a thin coat of primer (Ortho Solo, Ormco, Orange, CA, USA) and light cured orthodontic adhesive (Grengloo, Ormco, Orange, CA, USA). Excess adhesive was removed, and light curing was conducted from the mesial and distal side of the brackets (Bluephase 2.0i, Ivoclar Vivadent, Schaan, Liechtenstein; 10 s, 2 mm distance). All specimens were then subjected to thermocycling (THE-1100, SD Mechatronik GmbH, Feldkirchen-Westerham, Germany; 5 °C/55 °C, 10,000 thermal cycles, 20 s dwell time).

The shear bond strength of each specimen was measured using a universal testing machine (Zwick BZ050/TH3A, ZwickRoell, Ulm, Germany; occlusal force direction, crosshead speed 1 mm/min).

Numerical data were calculated as mean and standard deviation (SD). Shapiro–Wilk’s test was used to test for normality; homogeneity of variances was tested using Levene’s test. Welch one-way analysis of variance (ANOVA) test, followed by Games–Howell post hoc test were used for intergroup comparison. The significance level was set at *p* < 0.05 for all tests. Statistical analysis was performed using R software version 4.0.2 [18].

The study, including the use and CAP treatment of extracted human teeth, was approved by the Scientific Ethics Committee (Reg.-No. BB61/11b).

## Results

### Prestudy: Illustrating the morphological effects caused by different CAP compositions

**Fig. 1:**
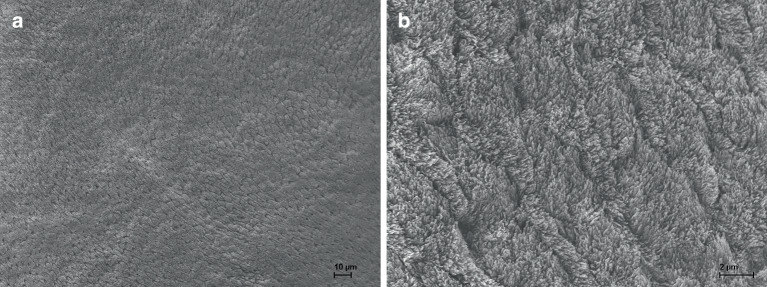
Representative scanning electron microscope (SEM) images, conditioning method: 35% phosphoric acid, 30 s, magnification: **a** 500 ×, **b** 5000 × Repräsentative elektronenmikroskopische Aufnahmen, Konditionierungsmethode: 35%ige Phosphorsäure, 30 s, Vergrößerung: **a** 500:1, **b** 5000:1

**Fig. 2:**
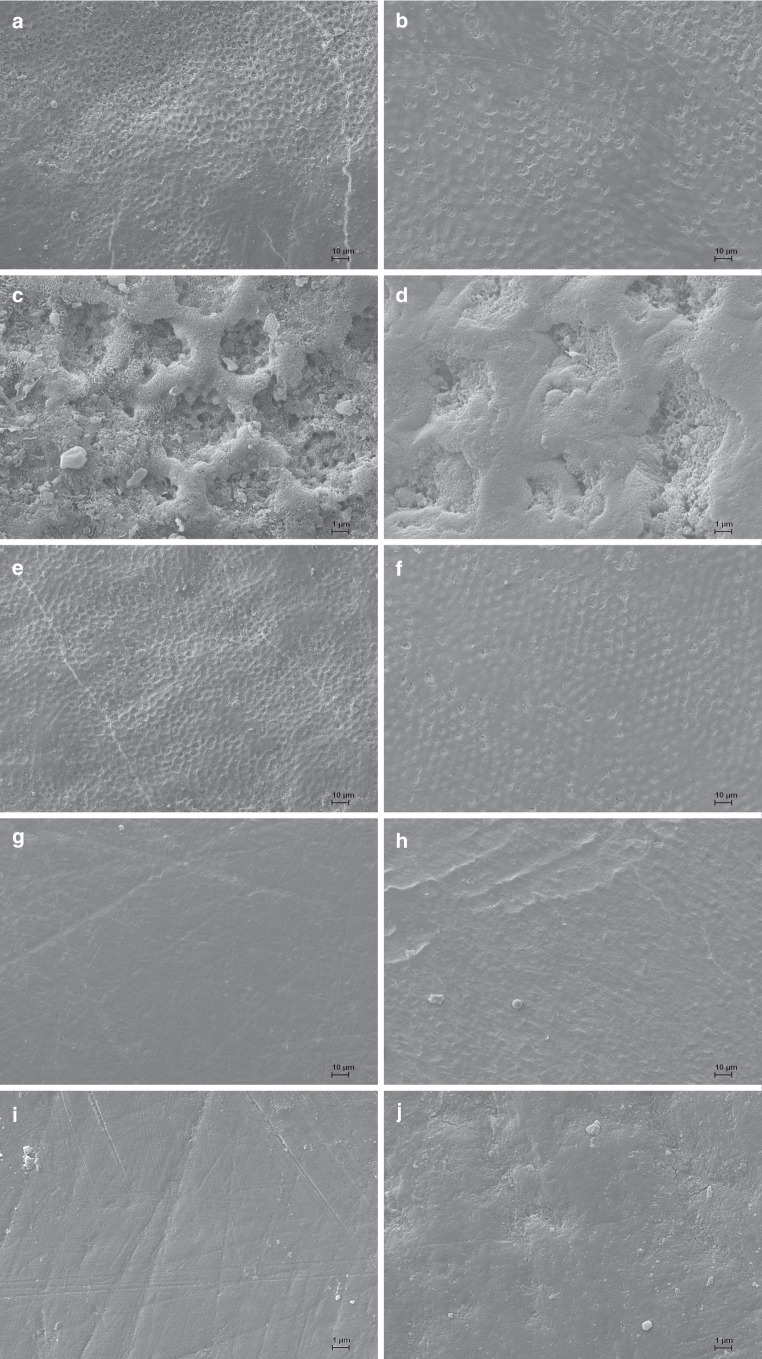
Representative scanning electron microscope (SEM) images: **a** bovine enamel, conditioning method: pure argon cold atmospheric plasma (CAP), 30 s, magnification: 500 ×, **b** human enamel, conditioning method: pure argon CAP, 30 s, magnification: 500 ×, **c** bovine enamel, conditioning method: pure argon CAP, 30 s, magnification: 5000 ×, **d** human enamel, conditioning method: pure argon CAP, 30 s, magnification: 5000 ×, **e** bovine enamel, conditioning method: argon CAP, 0.5% admixture of oxygen, 30 s, magnification: 500 ×, **f** human enamel, conditioning method: argon CAP, 0.5% admixture of oxygen, 30 s, magnification: 500 ×, **g** bovine enamel, conditioning method: argon CAP, 1% admixture of oxygen, 30 s, magnification: 500 ×, **h** human enamel, conditioning method: argon CAP, 1% admixture of oxygen, 30 s, magnification: 500 ×, **i** bovine enamel, untreated, magnification: 5000 ×, **j** human enamel, untreated, magnification: 5000 × Repräsentative elektronenmikroskopische Aufnahmen. **a** Boviner Zahnschmelz, Konditionierungsmethode: reines Argonplasma, 30 s, Vergrößerung: 500:1. **b** Humaner Zahnschmelz, Konditionierungsmethode: reines Argonplasma, 30 s, Vergrößerung: 500:1. **c** Boviner Zahnschmelz, Konditionierungsmethode: reines Argonplasma, 30 s, Vergrößerung: 5000:1. **d** Humaner Zahnschmelz, Konditionierungsmethode: reines Argonplasma, 30 s, Vergrößerung: 5000:1. **e** Boviner Zahnschmelz, Konditionierungsmethode: Argonplasma mit 0,5 % Sauerstoffbeimischung, 30 s, Vergrößerung: 500:1. **f** Humaner Zahnschmelz, Konditionierungsmethode: Argonplasma mit 0,5 % Sauerstoffbeimischung, 30 s, Vergrößerung: 500:1. **g **Boviner Zahnschmelz, Konditionierungsmethode: Argonplasma mit 1 % Sauerstoffbeimischung, 30 s, Vergrößerung: 500:1. **h **Humaner Zahnschmelz, Konditionierungsmethode: Argonplasma mit 1 % Sauerstoffbeimischung, 30 s, Vergrößerung: 500:1. **i **Boviner Zahnschmelz, unbehandelt, Vergrößerung: 5000:1. **j **Humaner Zahnschmelz, unbehandelt, Vergrößerung: 5000:1

The representative scanning electron microscopy (SEM) images illustrate how differently the conditioning methods affected the surface morphology of the enamel:

- 1) Etching with 35% phosphoric acid resulted in the substantial change and degradation of the enamel surface structure, exposing the prismatic structures with microporosities along the enamel crystallites (Fig. 1).
- 2) After the application of pure argon CAP, specimens showed surface alterations like shallow grooves, resulting from the differential dissolution of enamel prisms among prismatic and aprismatic enamel (Fig. 2a–d).
- 3) The application of argon CAP with 0.5% oxygen admixture resulted in shallow grooves with decreased quantity, distribution and intensity compared with enamel treated with pure argon CAP (Fig. 2e,f).
- 4) After the application of argon CAP with 1% admixture of oxygen, the enamel surface appeared unaffected and smooth (Fig. 2g,h).
- 5) Polished, untreated enamel showed a smooth surface (Fig. 2i,j).

In all groups, human and bovine enamel showed comparable morphological reactions to the conditioning methods, including the different compositions of argon CAP.

Oxygen admixture decreased the length of the plasma effluent, complicating precise application: with an admixture of 1% oxygen, the plasma effluent of kINPen MED was about 3 mm long, compared with 13 mm for pure argon CAP.

These observations led to the following conclusions for the study protocol of our main study:

- In this preclinical study, bovine enamel specimens can be substituted for human enamel specimens to gain more insight into CAP-based enamel conditioning.
- We chose not to increase the oxygen admixture to more than 0.5% in our main study to ensure good visibility and precise application of the plasma effluent.

### Main study: Determining the shear bond strength

The highest shear bond strength was found in specimens conditioned with argon CAP plus 0.5% oxygen that have been rewetted after CAP application (4.96 ± 1.00 MPa; group V), followed by the specimens conditioned with conventional acid etching (3.53 ± 0.71 MPa; group I). Specimens without any enamel conditioning show the lowest shear bond strength (0.24 ± 0.14 MPa; group II; Table 1).

**Table 1:** Descriptive statistics and normality test results for untransformed shear bond strength values (MPa) Deskriptive Statistik und Normalitätstestergebnisse für nichttransformierte Werte der Scherhaftfestigkeit (MPa)

Group	*n*	Mean	95% CI		SD	Median	IQR	Shapiro–Wilk	
			Lower	Upper				Statistic	*p*-value
I	25	3.53	3.25	3.81	0.71	3.47	0.70	0.894	0.093 ns
II	14	0.24	0.16	0.31	0.14	0.20	0.12	0.848	0.021*
III	25	1.73	1.28	2.18	1.15	1.39	1.46	0.930	0.305 ns
IV	25	2.16	1.81	2.51	0.90	2.15	1.22	0.951	0.581 ns
V	25	4.96	4.57	5.36	1.00	4.94	1.20	0.921	0.229 ns

*95%CI* 95% confidence interval for the mean, *SD* standard deviation, *IQR* interquartile range, *ns* not significant (*p* ≥ 0.05)

*significant (*p* < 0.05)

The assumption of normal distribution of the data was found to be violated in group II (*p* = 0.021); histograms of distribution showed positive skewness in all groups except group I. The data were log-transformed to achieve normality (Table 2). Levene’s test of homogeneity was found to be statistically significant (*p* < 0.001) indicating variance inequality between groups.

**Table 2:** Descriptive statistics and normality test results for log-transformed shear bond strength values (MPa) Deskriptive Statistik und Normalitätstestergebnisse für logarithmisch transformierte Werte der Scherhaftfestigkeit (MPa)

Group	*n*	Mean	95% CI		SD	Median	IQR	Shapiro–Wilk	
			Lower	Upper				Statistic	*p*-value
I	25	0.54	0.51	0.57	0.09	0.54	0.09	0.912	0.167 ns
II	14	−0.69	−0.81	−0.56	0.24	−0.71	0.26	0.964	0.793 ns
III	25	0.14	0.03	0.26	0.30	0.14	0.43	0.970	0.874 ns
IV	25	0.30	0.23	0.37	0.18	0.33	0.27	0.952	0.595 ns
V	25	0.69	0.65	0.72	0.09	0.69	0.11	0.930	0.303 ns

*95%CI* 95% confidence interval for the mean, *SD* standard deviation, *IQR* interquartile range, *ns* nonsignificant (*p* ≥ 0.05)

No significant difference was found between group III (pure argon CAP) and group IV (argon CAP plus 0.5% oxygen; *p* = 0.188). The differences between all other groups were statistically significant (*p* < 0.001; Tables 3 and 4).

**Table 3:** Mean and standard deviation values for log-transformed shear bond strength values (MPa) with robust analysis of variance (ANOVA) results Mittelwerte und Standardabweichung für logarithmisch transformierte Werte der Scherhaftfestigkeit (MPa) mit ANOVA(„analysis of variance“)-Ergebnissen

Mean ± SD					Statistic	Df1	Df2	*p*-value
Group I	Group II	Group III	Group IV	Group V				
0.54 ± 0.09^B^	−0.69 ± 0.24^D^	0.14 ± 0.30^C^	0.30 ± 0.18^C^	0.69 ± 0.09^A^	126.43	4	46.28	*<* 0.001*

Means with different superscript letters are statistically significantly different

*SD* standard deviation, *Df* Degree of freedom

*significant (*p* < 0.05)

**Table 4:** Results of Games–Howell post hoc test Ergebnisse des Games-Howell-Post-Hoc-Test

Groups	Mean difference	95% CI		Statistic	*p*-value
		Lower	Upper		
I–II	−1.22	−1.43	−1.02	18.678	*<* 0.001*
I–III	−0.39	−0.58	−0.21	6.333	*<* 0.001*
I–IV	−0.24	−0.35	−0.13	6.093	*<* 0.001*
I–V	0.15	0.08	0.22	5.901	*<* 0.001*
II–III	0.83	0.58	1.08	9.527	*<* 0.001*
II–IV	0.99	0.77	1.20	13.604	*<* 0.001*
II–V	1.37	1.17	1.58	20.861	*<* 0.001*
III–IV	0.16	−0.04	0.35	2.235	0.188 ns
III–V	0.54	0.36	0.72	8.674	*<* 0.001*
IV–V	0.39	0.27	0.50	9.764	*<* 0.001*

*95%CI* 95% confidence interval for the mean, *ns* nonsignificant (*p* ≥ 0.05)

*significant (*p* < 0.05)

## Discussion

Depending on the composition and protocol, the application of CAP led to significantly higher or significantly lower shear bond strength compared with conventional acid etching:

Brackets bonded on enamel that has been conditioned with argon CAP plus 0.5% oxygen and rewetted after CAP application (group V) yielded significantly higher shear bond strength than conventional acid etching (group I). Without rewetting (groups III and IV), the shear bond strength of specimens treated with argon CAP was significantly lower compared with conventional acid etching (group I), regardless of whether oxygen had been mixed to the feed gas or not.

Specimens that did not receive any conditioning (group II) showed particularly low shear bond strength, underlining the need for proper enamel conditioning: 11 of 25 specimens in this group showed spontaneous bracket failure during the thermocycling phase. Conditioning the enamel with argon CAP (groups III, IV, V) led to significantly higher shear bond strength compared with untreated enamel (group II), indicating that argon CAP does induce beneficial surface modifications for orthodontic bonding.

CAP application generates reactive species on the enamel surface through atom/ion bombardment [6], facilitating a chemical and physical bond between the enamel surface and the orthodontic bracket. The comparison of the scanning electron micrographs (Fig. 2a–h) indicates that argon CAP maintains the structural integrity of the enamel by removing organic sheaths of enamel prisms [12] without creating microporosities for the adhesive primer to infiltrate into. Almoammar et al. [1] reported that in the assessment of bond failure sites, CAP-conditioned specimens showed mainly cohesive failures, meaning that no or less than half of the adhesive remained on the enamel after debonding. Specimens conditioned with the acid etching method showed mainly adhesive failures with all or more than half of the adhesive left on the enamel. These findings support the assumption that CAP is gentler on the enamel than conventional acid etching. According to Marshall et al. [13] though, mechanical bonding—as it results from acid etching—is the primary factor for dental adhesion. This may explain the relatively lower shear bond strength on enamel conditioned with pure argon CAP (group III) and with argon CAP plus 0.5% oxygen without rewetting (group IV) compared with conventional acid etching.

CAP was applied using a commercial plasma source, the kINPen MED: a pen-sized, CE marked plasma jet [20]. Pen-to-sample distance was adjusted with a holding device to ensure the tip of the effluent touches the enamel surface. The kINPen MED plasma jet has a treatment field of approximately 1 cm^2^. We based the application time on the findings of Chen et al. [6] who described near super hydrophilicity after 30 s of treatment. Also, practitioners and patients are accustomed to similar conditioning times for conventional acid etch techniques, implicating practical feasibility in terms of time commitment.

### Oxygen admixture

Admixing oxygen to CAP prompts the formation of reactive oxygen species and hydrophilic C–O bonds on the treated enamel, thereby increasing the surface activity and chemical bonding properties [12]. In our study, the admixture of 0.5% oxygen (group IV) did increase the shear bond strength compared with pure argon CAP (group III), but this difference did not reach statistical significance.

Despite the lack of statistical difference in the functionalization ability, the admixture of 0.5% oxygen is preferable to pure argon CAP, because the scanning electron micrographs indicate that the extent of visible morphological effects decreased with the admixture of oxygen (Fig. 2a–f). Reuter et al. [19] found an inverse correlation of bullet velocity and oxygen content of CAP. Asghar et al. [2] and Bekeschus et al. [3] reported that the admixture of oxygen lowered the discharge plasma power. These findings underline our observation that oxygen admixture leads to less morphological alteration of the enamel. From a practical point of view, an admixture of 0.5% oxygen seems to be a good compromise between visibility of the plasma effluent, substance conservation, and functionalization.

### Rewetting after CAP application

Specimens in group V were rewetted with deionized water after the stream of argon CAP plus 0.5% oxygen had dried out the enamel surfaces. The shear bond strength in this group was significantly higher than in all other groups—including the conventional acid etching group (group I). According to Dong et al. [7], the process of water rinsing after CAP application helped to remove dislodged contaminants, thus, enhancing adhesive penetration. We used deionized water to prevent the suppression of reactive ions on the enamel surface. The significant difference between group IV (2.16 ± 0.90 MPa) and group V (4.96 ± 1.00 MPa) shows the importance and efficiency of rewetting.

Excessive shear bond strength can cause enamel damage when brackets are removed. However, we did not find any signs of enamel cracks or hard substance loss on the specimens after debonding—not even in group V that showed higher shear bond strength values than the conventional acid etching group.

### Study limitations

While in vitro studies cannot fully imitate intraoral conditions like masticatory forces, moisture, and temperature, thermocycling is an option to simulate these influences on bond strength as close as possible [10]. Our protocol aimed to simulate one year of function.

The mean shear bond strength for conventional acid etching (group I) and CAP application (group IV) was lower than in other studies [1, 4], probably due to effects of thermocycling and the type of specimen. Bovine enamel, as used in our study, is an appropriate substitute for human enamel in adhesion studies [9], but tends to show lower shear bond strength [15, 16]. In their systematic review on in vitro orthodontic bond strength testing, Finnema et al. [8] question the validity of thresholds for clinically acceptable bond strength values. The interpretation of our results therefore focuses on the relative effectiveness of conditioning protocols and not on the comparison of absolute values with thresholds or with other studies.

## Conclusion

In this experimental setting, the admixture of 0.5% oxygen and the rewetting of the enamel after cold atmospheric plasma (CAP) application have emerged as important steps for CAP conditioning prior to bracket bonding. Based on this composition and protocol, CAP could represent a gentle alternative to acid etching in orthodontic bonding.
